# Identification and quantification of novel RNA isoforms in horn cancer of *Bos indicus* by comprehensive RNA-Seq

**DOI:** 10.1007/s13205-016-0577-5

**Published:** 2016-12-07

**Authors:** Subhash J. Jakhesara, Prakash G. Koringa, Neelam M. Nathani, Chaitanya G. Joshi

**Affiliations:** Department of Animal Biotechnology, College of Veterinary Science and Animal Husbandry, Anand Agricultural University, Anand, Gujarat 388 001 India

**Keywords:** Horn cancer, Alternative splicing, GS-FLX Titanium, Transcriptome profiling, Splice isoform

## Abstract

Horn cancer (HC) is a squamous cell carcinoma of horn, commonly observed in *Bos indicus* of the Asian countries. To elucidate the complexity of alternative splicing present in the HC, high-throughput sequencing and analysis of HC and matching horn normal (HN) tissue were carried out. A total of 535,067 and 849,077 reads were analysed after stringent quality filtering for HN and HC, respectively. Cufflinks pipeline for transcriptome analysis revealed 4786 novel splice isoforms comprising 2432 exclusively in HC, 2055 exclusively in HN and 298 in both the conditions. Based on pathway clustering and in silico verification, 102 novel splice isoforms were selected and further analysed with respect to change in protein sequence using Blastp. Finally, fourteen novel splicing events supported both by Cufflinks and UCSC genome browser were selected and confirmed expression by RT-qPCR. Future studies targeted at in-depth characterization of these potential candidate splice isoforms might be helpful in the development of relevant biomarkers for early diagnosis of HC. The results reported in this study refine the available information on transcriptome repertoire of bovine species and boost the research in the line of development of relevant biomarkers for early diagnosis of HC.

**Electronic supplementary material:**

The online version of this article (doi:10.1007/s13205-016-0577-5) contains supplementary material, which is available to authorized users.

## Introduction

Cancer is a multifactorial disease affecting almost every species of animals and human. Various types of cancers are prevalent in farm animals causing moderate to severe economic losses. Horn cancer (HC, cancerous tissue from affected horn) is amongst widely prevalent cancer reported in Indian zebu cattle (*Bos indicus*), drawing recent attention towards the necessity of in-depth studies for better understanding. HC is a progressive cancer which is classified into three different stages based on the growth of tumour tissue in the affected horn. The incidence of HC in India is more commonly seen in Kankrej breed than other zebu and crossbred cattle (Joshi et al. 2009; Udharwar et al. 2008). Exceptional few cases of HC have been reported from other countries like Sumatra (Burggraaf 1935), Brazil (Rezende and Naves 1975) and Iraq (Zubaidy 1976). The mainly affected animals are Bullocks (95%) in comparison to cows (5%), whereas it is rarely reported in bulls, buffaloes, sheep and goats (Chattopadhyay et al. 1982; Damodaran et al. 1979; Gupta et al. 1980; Kulkarni 1953). HC is a squamous cell carcinoma of horn arising from pseudo-stratified columnar epithelium of the horn core mucosa. The main difficulties in early diagnosis include the absence of specific markers and the lack of a complete understanding of the cellular hierarchy of morphogenesis of the horn core epithelium. At molecular level, HC has been studied previously for novel gene content (Jakhesara et al. 2013b) and global gene expression (Tripathi et al. 2012).

Horn cancer involves complex interplay of several genes which are up-regulated or down-regulated and results in the transformation of normal horn tissue to malignant one. In horn cancer, the interplay of three genes *KRT6A*, *KRT6B*, and *KRT6C* has been observed in cancer. Over and above these keratin protein family members, there is also up-regulation of a novel candidate *KRT84*. Other genes of keratin family, *KRT14* and *KRT5*, are specifically expressed in the basal layer of the epidermis and found to be up-regulated in HC. Gene *KRT14* expression can be considered as a candidate of proliferative activity and metastatic potential of SCC in horn. Besides keratin family genes, Stratifin (*SFN*), Anilin (*ANLN*), *NR4A1* and *SCGB1A1* (Uteroglobin) were also significantly up-regulated in HC tissue. While, a number of genes were observed to be down-regulated in HC including *CXCL17*, *KRT19* and *BPIFB1* which are known to be involved in anti-tumour immune reactions. *CXCL17* has also been reported to have potential tumour suppressor activity (Hiraoka et al. 2011). *BPIFB1* is commonly known as long palate, lung and nasal epithelium carcinoma-associated protein 1 (*LPLUNC1*) expressed in the epithelium of upper respiratory tract (Bingle et al. 2010). Additional details about commonly expressed genes in HC are described elsewhere (Koringa et al. 2013).

Amongst diverse type of changes present in the cancer, overrepresentation of aberrant mRNA transcripts is the most common and widely reported observation (Jakhesara et al. 2013a; Li et al. 2013; Erho et al. 2012). Alternative splicing (AS) is the phenomenon by which the exons of primary transcripts are used alternatively to produce structurally and functionally distinct mRNA (splice isoforms) and protein variants, which might be one of the most extensively used mechanisms that accounts for the greater cellular complexity of higher eukaryotic organisms (Irimia and Blencowe 2012). Cancer cells may exhibit defective regulation of this alternative exon usage that can result in changes with potential transcriptome-wide consequences to gene function. It is imperative that AS can result in increased protein repertoire of the cell encoding alternative protein forms with biological functions that differ from the canonical product of the locus. AS transcripts exhibit changes to the primary transcript and might result in transcripts and/or protein isoforms with loss or disruption of domains responsible for original function.

Detailed investigation and understanding of complexity of AS events exhibited by cancer cells are very important as it provides important clues about structural and functional consequences of deregulated genes. Until recently, profound cost, limited coverage and sensitivity of the sequencing and microarray studies were hindering the global study of AS events in the cell; however, with advancement in the next-generation sequencing technologies now it is possible to do the same. Although, HC has been studied recently for detection of AS events, the information generated was on limited scale, with small size of dataset and analysis lacking RNA-Seq-specific pipeline (Patel et al. 2013). In the present study, we employed high-throughput pyro sequencing combined with Cufflinks workflow optimized for RNA-Seq to elucidate complexity of the AS in HC transcriptome. Here, we report AS events exhibited in HC and HN tissue and confirmed their significant association with HC by RT-qPCR.

## Materials and methods

### Tissue collection and RNA sequencing

All experimental protocols were performed as per guidelines and approval of Institutional Animal Ethics Committee of the Anand Agricultural University (Permit Number 486). Detailed summary of methods employed for tissue collection and sequencing is described elsewhere (Jakhesara et al. 2013b). Briefly, after mRNA isolation from collected tissues of HC and HN, cDNA libraries were prepared and sequenced on GS FLX Titanium following manufacturer protocol (Roche Diagnostics, Switzerland).

### Mapping of RNA-Seq reads using GMAP

After signal processing and trimming, available reads were aligned to the UCSC *Bos taurus* reference genome (build bosTau7) using GMAP v2012-06-06 (Wu and Watanabe 2005). Whenever required, we used SAM Tools v0.1.18 (Li et al. 2009) to convert SAM files to BAM file and vice versa. Mapping results were visualized using a local copy of the Integrative Genomics Viewer v 2.1.17 (Thorvaldsdottir et al. 2012) software available at http://www.broadinstitute.org/igv/.

### Transcript assembly using Cufflinks

The read alignments from GMAP were processed by Cufflinks v2.0.0 (Trapnell et al. 2010) for assembly of the reads into transcripts; their abundance estimation and tests for differential expression between the cancer and normal tissue samples were performed using Cufflinks. Cufflinks also uses reference annotation-based transcript (RABT) assembly method (Roberts et al. 2011), to assemble against a known reference annotation to better identify novel transcripts. Cufflinks measures transcript abundances in Fragments Per Kilobase of exon per Million fragments mapped (FPKM), which is analogous to single-read “RPKM”. Cufflinks was run with default parameters for RABT assembly method.

### Comparison to reference annotation and differential analysis

Once assembly of alignments of reads to transcripts is completed; the transcripts.gtf files from Cufflinks were used as input to Cuffcompare along with reference annotation file refFlat.gtf file downloaded from UCSC Genome Browser Database (build Btau7) (Dreszer et al. 2012). Combined.gtf file produced by Cuffcompare was provided as input to Cuffdiff along with alignment files produced by GMAP for differential analysis between two samples. Cuffcompare and Cuffdiff were run employing default parameters with refFlat.gtf file of *Bos taurus* genome Btau7 build for reference annotation.

### Functional analysis of gene isoforms

The Database for Annotation, Visualization and Integrated Discovery (DAVID) v6.7 is a set of web-based functional annotation tools for functional clustering of genes (Huang et al. 2009). The functional clustering tool was used for functional enrichment of isoforms with significant differential expression. Gene ontology was selected for functional annotation with Fishers exact test at threshold value of 0.001. Similarly, pathway analysis is also performed with DAVID employing Fishers exact test at a threshold value of 0.01.

### Validation of potential novel splices isoforms

The tracking file provided by Cuffcompare was used to infer splice isoforms unique to cancer by examining manually. The transcripts with class code *j* were selected for further evaluation. All novel isoforms detected by Cuffcompare were classified based on their uniqueness in case of HC, HN and present in both the cases. Further, these novel isoforms were subjected to pathway-based clustering using the highest classification stringency parameter in DAVID v6.7. From these, novel splice isoforms clustered under pathways associated with cancer were selected for further in silico verification with IGV for actual read support and BLAT program of UCSC genome browser for EST support. Transcripts assembled from the reads supporting these splice isoforms were translated into protein sequence for evaluation of protein change by comparison with reference protein sequence and loss of functional domain by blastp (Altschul et al. 1990). At last, fourteen transcripts showed novel splicing both by Cufflinks and UCSC genome browser and a significant change in protein sequence with loss of functional motif was confirmed by RT-PCR. The primers for RT-PCR were designed from exon–exon junction in such a way that it should flank the location of splice site change.

### Quantitative real-time PCR (qPCR)

Four samples each of HC and HN were used to isolate total RNA and differential expression of fourteen novel splice isoforms was confirmed through RT-qPCR. The FASTA sequences of reads spanning each selected transcript were obtained from IGV and queried using web based tool Primer-BLAST (Ye et al. 2012) against *Bos taurus* reference transcriptome to check the specificity of primers to amplify only isoform transcripts. The expression level of novel splice isoforms was quantified by RT-qPCR analysis on ABI PRISM 7500 fast real-time PCR system (Applied Biosystems) using Quantifast SYBR Green PCR master mix (Qiagen). For PCR, all reactions were run in triplicate and cycle threshold (*C*_T_) values for target novel transcripts were normalized with endogenous reference gene RPLP0. The fold change between two conditions was calculated using the formula as described here:

$${\text{Fold expression}} = 2^{{ - \Delta \Delta C_{\text{T}} }}$$

where Δ*C*_T_ is the average *C*_T_ of target isoform − average *C*_T_ of endogenous control (RPLP0), and ΔΔ*C*_T_ is the Δ*C*_T_ of target sample (HC) – Δ*C*_T_ of calibrator sample (HN).

## Results

### Deep sequencing of *Bos indicus* horn transcriptome

To fully characterize and identify HC transcriptome, three technical replicates of HC and HN sample were processed for sequencing in three different runs. Thereafter, we pooled sequencing data from all three technical replicates of each sample for further analysis. As a result of sequencing, we obtained a total of 0.59 million and 0.92 million high-quality reads comprising 239 million and 372 million nucleotide bases for HN and HC tissue, respectively. The reads used for analysis are submitted to the bioProject NCBI and publically available with Accession number PRJNA353983. Raw sequenced reads were processed for quality filtering on the basis of quality score and homopolymer runs. After quality filtering, we retained 535,067 and 849,077 reads, respectively, for HN and HC, for further mapping and analysis of digital gene expression profiling. We used GMAP software package for alignment of sequencing sequenced reads against *Bos taurus* reference genome (bTau7). Approximately, 94.8 and 94.3% of reads were successfully mapped to reference consisting of 80.3 and 79.7% of uniquely mapped reads, respectively, for HC and HN. Detailed summary of mapping is described elsewhere (Jakhesara et al. 2013b).

### Differentially expressed gene isoforms

**Fig. 1:**
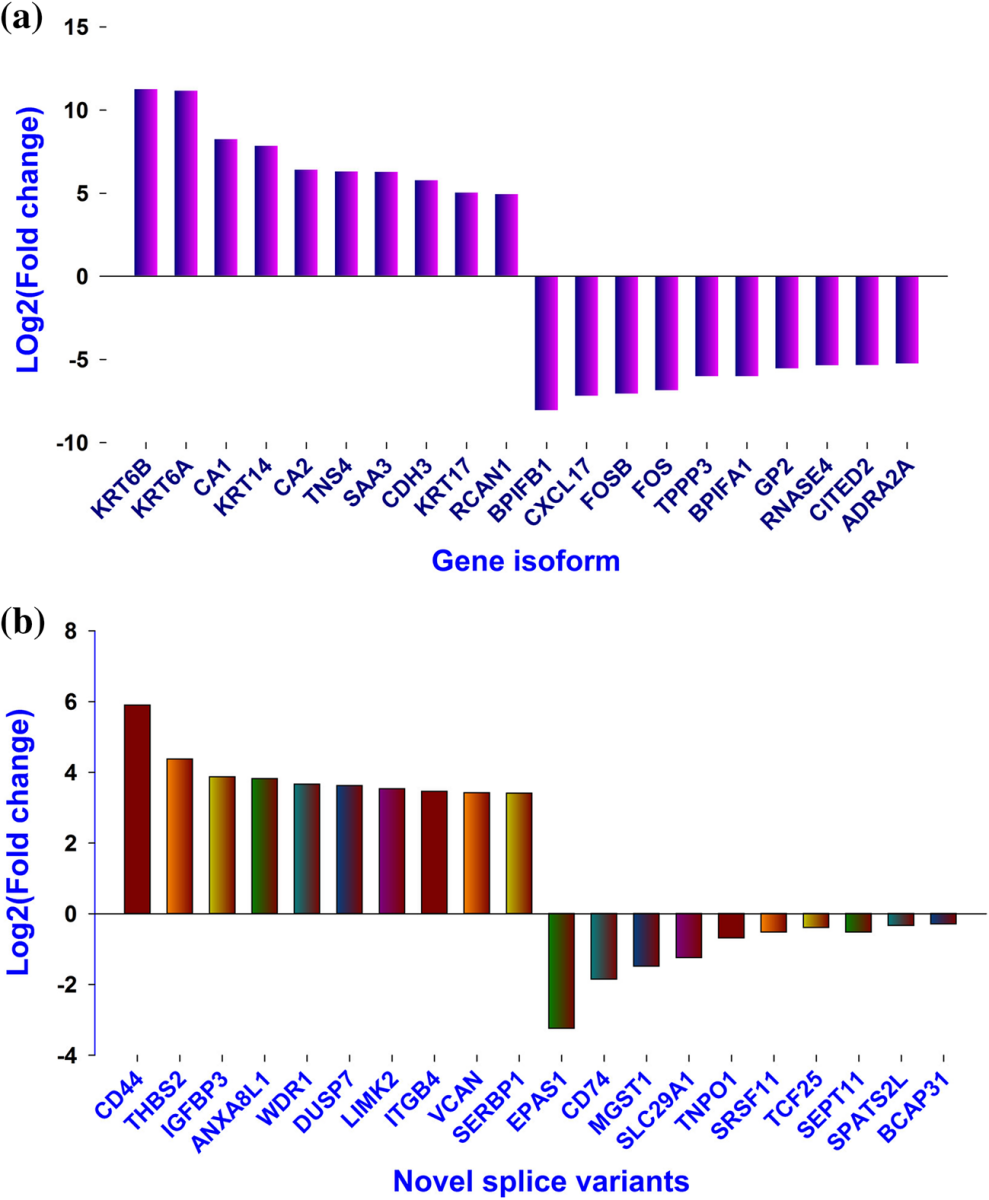
Top 10 up-/down-regulated isoforms in horn cancer. **a** Top 10 up-/down-regulated isoforms with significant differential expression in HC. The differentially expressed isoforms in HC vs. those in normal tissue were determined by Cuffdiff. The fold change is the ratio of FPKM of these isoforms in HC to FPKM in normal tissue. The differentially expressed isoforms were ranked on their fold change and 10 isoforms with the highest or lowest fold changes are shown in the figure. **b** Top 10 up-/down-regulated novel splice variants in HC. The fold change is the ratio of FPKM of these isoforms in HC to FPKM in normal tissue. The novel splice variants were ranked on their fold change and 10 splice variants with the highest or lowest fold changes are shown here

In total, 13,020 known isoforms were detected in HC while 12,728 known isoforms were detected in HN. Amongst these, 2739 isoforms were detected only in HC, while 2447 isoforms were detected only in HN. In total, 1252 known isoforms were up-regulated, while 1543 down-regulated isoforms were observed with at least twofold difference in HC tissue in comparison to HN. Top ten up- and down-regulated isoforms with significant differential expression are presented in Fig. 1a. More extensive lists of all 130 gene isoforms with significant differential expression in HC are given in Table ST1. The fold change amongst top 50 up-regulated isoforms was from 11 to 2.7; while in case of down-regulated isoforms, it was −8 to −2.5-fold.

### Functional annotation of isoforms with significant differential expression

All isoforms with differential expression were subjected to functional analyses for gene ontology and pathway analysis. The results revealed significant enrichment of cancer (9.63E−04) and colorectal cancer (5.80E−03) pathways. Gene ontology analyses also revealed enrichment of several biological processes at threshold of 0.001. Mainly, biological processes associated with angiogenesis, epithelial cell development and differentiation, cell proliferation and cell death were found to be enriched in differentially expressed genes. Table 1 shows list of biological processes and pathways enriched in differentially expressed isoforms.

**Table 1:** List of pathways and functional categories deregulated in isoforms with significant differential expression in HC

Pathway	Term	*P* value
*Pathways deregulated in HC*		
KEGG_PATHWAY	bta05200:Pathways in cancer	9.63E−04
KEGG_PATHWAY	bta05210:Colorectal cancer	5.80E−03
KEGG_PATHWAY	bta04010:MAPK signalling pathway	2.26E−02
KEGG_PATHWAY	bta04610:Complement and coagulation cascades	2.33E−02
KEGG_PATHWAY	bta00680:Methane metabolism	5.11E−02
*Gene ontology categories deregulated in HC*		
GOTERM_BP_FAT	GO:0001944~vasculature development	1.39E−07
GOTERM_BP_FAT	GO:0001568~blood vessel development	1.21E−06
GOTERM_BP_FAT	GO:0048514~blood vessel morphogenesis	3.20E−06
GOTERM_BP_FAT	GO:0001525~angiogenesis	2.98E−05
GOTERM_BP_FAT	GO:0008285~negative regulation of cell proliferation	6.46E−05
GOTERM_BP_FAT	GO:0060429~epithelium development	1.02E−04
GOTERM_MF_FAT	GO:0003700~transcription factor activity	2.36E−04
GOTERM_BP_FAT	GO:0035295~tube development	2.73E−04
GOTERM_BP_FAT	GO:0006357~regulation of transcription from RNA polymerase II promoter	4.24E−04
GOTERM_BP_FAT	GO:0010604~positive regulation of macromolecule metabolic process	4.64E−04
GOTERM_BP_FAT	GO:0030855~epithelial cell differentiation	6.88E−04
GOTERM_BP_FAT	GO:0008219~cell death	7.15E−04
GOTERM_BP_FAT	GO:0010628~positive regulation of gene expression	7.84E−04
GOTERM_BP_FAT	GO:0016265~death	8.32E−04
GOTERM_BP_FAT	GO:0009967~positive regulation of signal transduction	9.19E−04

### Novel splice isoforms and their validation

Cuffcompare detected several transcripts with novel splicing and all detected transcripts were classified as intergenic, complete match, novel or unknown. A total of 4785 novel splice isoforms were detected by Cuffcompare, which included 2432 isoforms unique for HC, 2055 isoforms unique for HN and 298 splice isoforms were observed in both the conditions. Figure 1b shows top 10 novel up- and down-regulated splice isoforms in HC and HN sample.

**Fig. 2:**
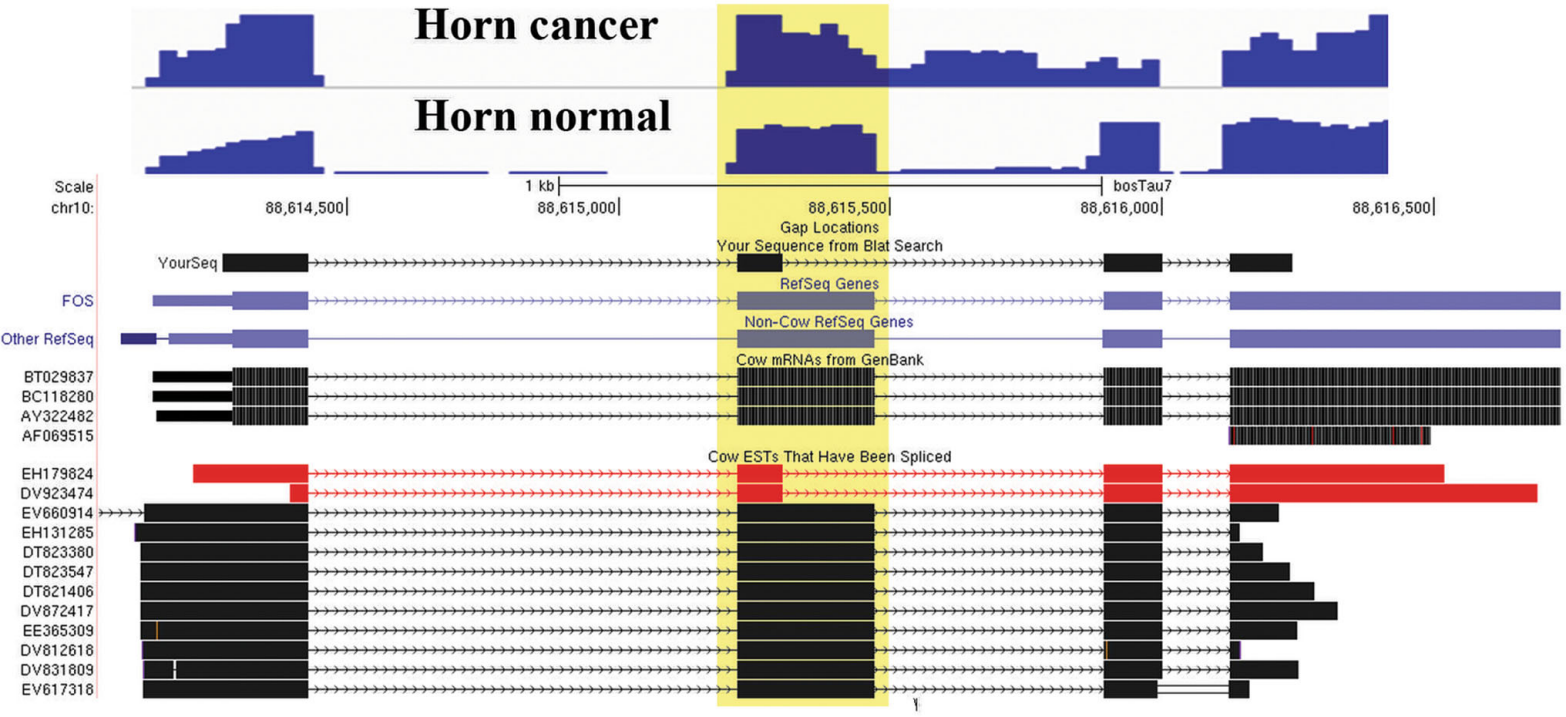
Representative figure of typical novel splice variant identified in our study. Figure was reconstructed using images from IGV and UCSC genome browsers. At the *top* of the figure (*blue peaks*) coverage tracks from IGV genome viewer are shown, while the diagrammatic representation showing transcript structures for FOS gene was taken from UCSC genome browser after BLAT analysis of reads showing novel splice variation. *Black blocks* show exonic regions, while *regions with arrows* indicate intronic regions. ESTs supporting the splice variation are shown in *red*. *The region marked with faint yellow block* shows the exon with splice change

The large number of novel splice isoforms was found in the present study; hence, to select the events relevant to the cancer, KEGG pathway analyses and gene ontology analyses were performed. Splice isoforms uniquely present in HC and HN as well as common isoforms in both conditions were subjected to KEGG pathway analyses using DAVID tool employing highest sensitivity. As a result of this analysis, three clusters (cancer-related pathways, cardiomyopathy and amino acid metabolism) were found to be enriched in HC and HN. Based on this analysis and in silico verification with IGV, respectively, 42, 43 and 17 splice isoforms were selected for HC, HN and common to both conditions (Table ST2). Figure 2 shows representative image of one such novel splice isoform detected in FOS gene.

All splice isoforms were evaluated with blastp for protein change and details are available as Table ST2. Finally, 14 transcripts (7 isoforms uniquely expressed in HC, 5 transcripts uniquely expressed in HN and 2 isoforms present in both) were selected for RT-qPCR verification based on isoforms showing frame shift, loss of active domain and association with cancer. Table 2 shows list of splice isoforms with type of variation selected for RT-qPCR. Primers designed from exon–exon junction for amplification of these reference and variant transcript are given in Table ST3.

**Table 2:** List of splice variants selected for validation along with type of variation and protein change

Gene	Accession no. of reference gene	EST support	Type of variation	Chr.	Protein change
FOS	NM_182786.2	Yes	Alternate 3′ splicing exon 2	10	Premature stop codon after 104 aa
MAPK9	NM_001046369.1	Yes	Alternate 5′ splicing exon 10	7	Premature stop codon after 293 aa
FGFR1	NM_001110207.1	No	Skipping of exon 2, alternate 3′ splicing exon 3	27	Deletion of aa 31–117, loss of Ig1_FGFR domain
FGFR2	NM_001205310.1	No	Skipping of exon 10–13	26	Deletion of aa 448–639, loss of PTKc_FGFR domain
MFAP4	NM_001080217.1	No	skipping of exon 2–5, alternate 5′ splicing exon 6	19	Premature stop codon after 157 aa, part of fibrinogen related domain lost
HSP90AB1	NM_001079637.1	Yes	Intron retention between exon 7–8	23	Premature stop codon after 380 aa, HSP90 domain lost
RPS6KB1	NM_205816.1	No	Alternative 3′ splicing exon 7	19	Premature stop codon after 234 aa, part of STKc domain lost
CREBBP	NM_001164022.1	No	Alternate 3′ splicing exon 9	25	Premature stop codon after 649 aa, Creb domain lost
CRK	NM_001192334.1	Yes	Novel exon between 1–2, alternate 3′ splicing of exon 2	19	Premature stop codon after 204 aa, SH3 domain lost
PTPN6	NM_001098017.1	No	Skipping of exon 12	5	Premature stop codon after 457 aa, part of PTPc domain lost
PTPRU	NM_001191494.1	No	Alternate 5′ splicing exon 7	2	Premature stop codon after 312 aa, several domains of fibronectine and PTPc are lost
SUFU	NM_001098083.2	No	Alternate exon 11	26	Same size of protein with different terminal 52 aa, different C terminal domain than ref.
FN1	NM_001163778.1	No	Skipping of exon 32	2	Deletion of 1631–1720 aa without frame shift
FN1	NM_001163778.1	Yes	Novel exon 25	2	Addition of 91 aa after position 1266 without frame shift

**Fig. 3:**
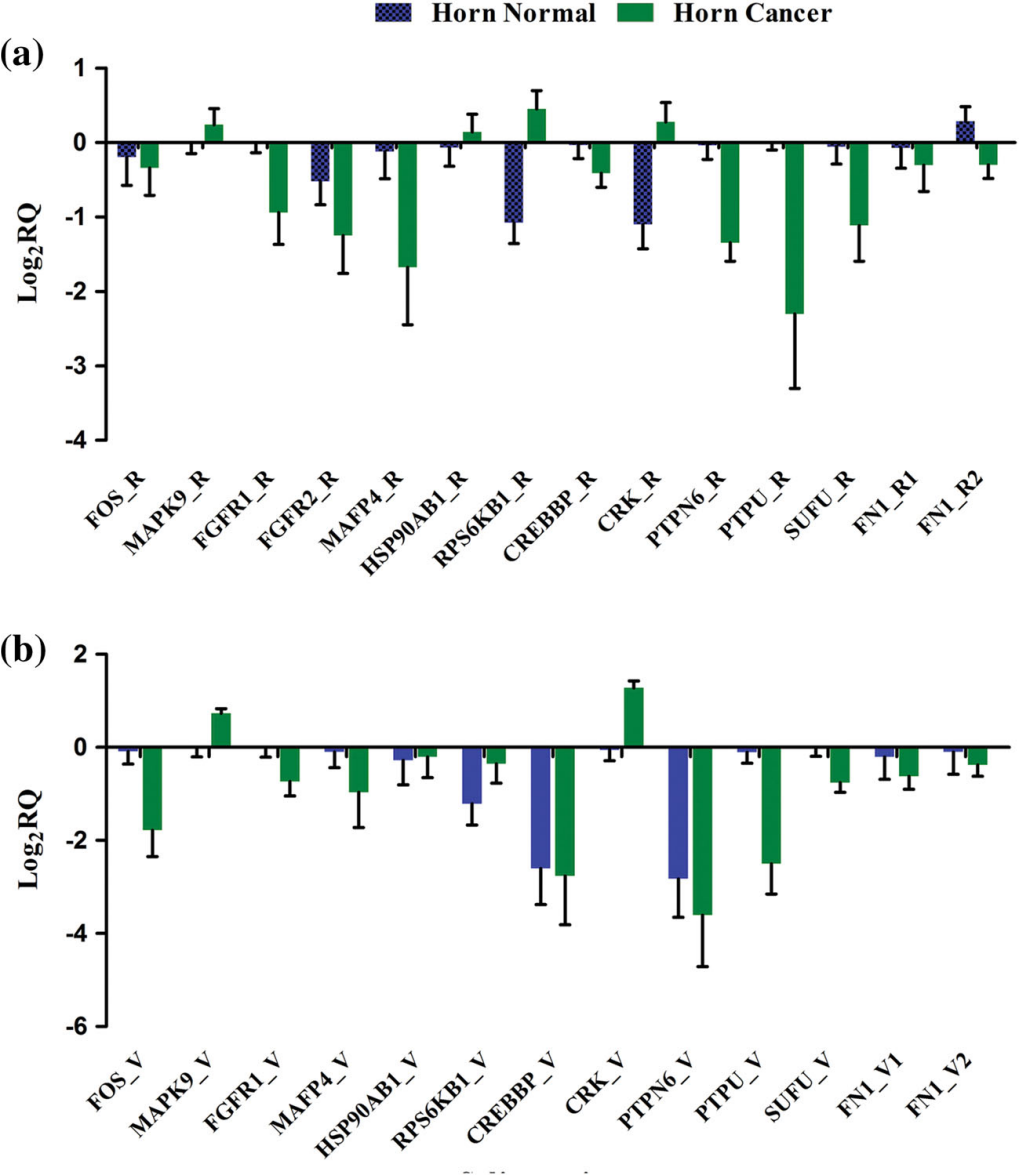
RT-qPCR analysis of novel splicing events. RT-qPCR analysis of novel splicing events on multiple horn cancer samples. The relative expression of reference (**a**) and variant (**b**) isoforms of candidate genes was analysed in four horn cancer tissue samples relative to four normal horn tissues. Gene names indicated below are the columns for respective genes, where _R and _V following gene names indicate reference and variant isoforms, respectively. The logarithmic transformation of relative quantitation (log_2_RQ) of each candidate isoform in cancer tissues was plotted against their expression in normal tissue. *Error bar* indicates standard error of mean (SEM) of four biological replicates of HC or HN

The expression profile of 14 novel splice events and the corresponding reference transcripts obtained with RT-qPCR is presented in Fig. 3. The RT-qPCR analysis of HC and HN tissue confirmed the expression of all reference as well as novel splice isoforms except FGFR2 isoform, which could not be amplified after repeated attempts on different samples. However, we found some discrepancies between RT-qPCR and RNA-Seq expression results. RT-qPCR revealed down-regulation of all the splice isoforms which were found to be up-regulated by RNA-Seq except MAPK9, while in case of down-regulated isoforms complete correlation was observed between RT-qPCR and RNA-Seq.

## Discussion

Cancer results from culmination of variety of genetic and epigenetic changes occurring over a period of time. In such a disease, it would be very much helpful to examine molecular signatures that differentiate normal cell from cancer one, as it will unravel nature and pathologic behaviour of a cancer cell. Here, we have made an attempt to unravel the novel splice isoforms expressed in horn cancer as a result of complex RNA editing events through high-throughput transcriptome analyses of horn cancer and its normal counterpart. We found and validated several splice isoforms expressed in horn cancer that might play a role in progression of horn cancer. Our results show that high-throughput transcriptome sequencing along with cautious in silico and wet lab validation approaches can identify novel splice isoforms associated with cancer.

Our analyses with Cuffdiff for differential expression revealed several differentially expressed known isoforms in HC. Many of the gene isoforms identified are known to be involved in varieties of cancers; however, it would be important to discuss top deregulated and differentially expressed known gene isoforms in the present study. Amongst top up-regulated isoforms, KRT gene isoforms (*KRT6A*-11 fold, *KRT6B*-11 fold, *KRT14*-5 fold and *KRT17*-5 fold) are expressed during differentiation of simple and stratified epithelial tissues, which justify their overexpression in horn cancer. These genes are also reported for overexpression in squamous cell carcinoma of lung (Hawthorn et al. 2006; Ohkura et al. 2005; Xue et al. 2011). At the second place, isoforms of *CA1* (8-fold) and *CA2* (6.5-fold) genes were significantly overexpressed in HC. Both the gene isoforms are implicated in more advanced stage of the oral squamous cell carcinoma but not with metastasis (Liu et al. 2012). These isoforms might serve as a prospective biomarker to predict growth and metastasis of tumour cells. Next up-regulated isoform *TNS4* variant (*CTEN*) is a member of family of proteins that are localized to integrin-linked focal adhesions. *CTEN* is implicated for increasing invasiveness through colony formation and anchorage-independent growth in colorectal, breast, gastric and pancreatic cancers (Liao et al. 2009; Sakashita et al. 2008; Albasri et al. 2011; Al-Ghamdi et al. 2013). SAA3 isoform which encodes for serum amyloid A protein has been widely studied as a marker in inflammation. Though its association in cancer is yet to be studied, it is observed that it might play a role in pre-metastatic stage and increases migration of metastatic cells to lungs (Hiratsuka et al. 2008). This observation makes *SAA3* a plausible candidate target gene to track and prevent metastasis. *CDH3* isoform encoding for adhesion glycoprotein is a member of cadherin family responsible for cell–cell adhesion and reported for its overexpression in several cancers (Paredes et al. 2012). *RCAN1* isoform encodes for protein regulator of calcineurin 1 and its overexpression inhibits migration of cancer cells (Espinosa et al. 2009). Amongst top down-regulated isoforms, *BPIFB1* (8-fold) and *BPIFA1* (6-fold) are members of a family of secreted proteins with unclear biological role and function, but it has been reported that these proteins are differentially expressed in lung diseases (Bingle et al. 2012). *CXCL17* is a latest member of the C-X-C chemokine family proteins and aberrant expression of it promotes tumour progression through angiogenesis (Matsui et al. 2012). Next down-regulated isoforms *FOS* and *FOSB* (7-fold) are members of FOS family of proteins involved in the regulation of cell proliferation, differentiation, and transformation. These proteins are extensively studied and their down-regulation is shown to promote tumour progression and metastasis (Kim et al. 2010). *TPPP3* isoform, also known as p20, is a new member of the tubulin polymerization promoting protein (TPPP) family playing a role in microtubule assembly. TPPP3 protein is not reported in cancer, but its knockdown suppressed cell proliferation and induced cell cycle arrest in HeLa cells (Zhou et al. 2010). Three isoforms *GP2*, *RNASE4* and *ADRA2A* amongst top down-regulated genes are not reported to be differentially expressed in cancer. *CITED2* isoform encoding for Cbp/P300-Interacting Transactivator protein plays an important role in cell proliferation by responding to TGF-α induction and TGF-β suppression to orchestrate cellular proliferation and quiescence, respectively. It is also reported that *CITED2* knockdown caused tumour shrinkage and increased overall host mouse survival rates (Chou et al. 2012).

Gene ontology and pathway analyses of differentially expressed isoforms revealed important insights about the predominantly affected functions in HC. Gene ontology suggested that HC is an actively growing tumour with changes involving epithelial cell differentiation and epithelium development with angiogenesis. Pathway analyses suggested that cancer pathways and MAPK signalling pathway responsible for uncontrolled cell growth are differentially enriched in HC. Further in-depth analysis of genes involved in MAPK/ERK signalling pathway is necessary to support and confirm their role in uncontrolled cell growth. Pathway analysis also resulted in clustering of pathways related to cardiomyopathy. Investigation of the genes (ACTN1, ACTN4, ADCY6, CTNNB1, DSP, EMD, ITGA2, ITGA5, ITGAV, ITGB1, ITGB3, ITGB5, JUP, PRKAA1, TPM1, TPM2, TPM4, and TCF7) clustered under the cardiomyopathy pathways revealed involvement of these genes in calcium binding pathways. As, calcium binding is important for normal functioning of heart through binding with troponins, enrichment of these genes resulted in the enrichment of cardiomyopathy pathways (Farah and Reinach 1995).

Our analyses with Cuffcompare detected several transcripts with novel splicing amongst all detected transcripts classified as intergenic, complete match, novel or unknown. To reduce these novel splicing events to more meaningful conclusion and to reduce number of false positives, we employed rigorous bioinformatics analysis. As a first step, we reduced these numbers by selecting only those novel splice events which were clustered along with cancer pathways in pathway analysis; this helped us to select splicing events which might be associated with HC. Second, we analysed all selected splicing events by UCSC genome browser, BLAT and IGV to check for EST support, to verify novel splicing and to eliminate splicing events not supported by reads, respectively. We also eliminated the events which were present at the end of the reads. As a third and final step, we performed blastp of the translated protein sequences of novel splices transcripts to examine amino acid changes in the sequence of the protein. In this step, we selected only splicing events which resulted in significant protein change or premature stop codon. Eventually, we selected 14 novel splicing events and confirmed their presence by end-point PCR and by RT-qPCR. This intensive selection and filtering of novel splicing events made sure that only splice isoforms which might be significantly associated with HC are analysed.

Our validation of selected novel splicing events revealed discrepancy in the expression profiles obtained by RT-qPCR with that of RNA-Seq. The contrasting differential expression profile obtained with RT-qPCR in few isoforms reveals the limitation of RNA-Seq to identify reads originating from specific isoform and therefore requires additional validation using RT-qPCR for changes in gene expression, while confirmation of almost all splicing events by RT-qPCR denotes reliability of RNA-Seq for high throughput detection of novel splicing events. Additionally, differential expression of all in silico selected splice isoforms across multiple cancer samples validates our approach for selection of novel splice isoforms relevant to cancer. We found a consistent pattern of down-regulation or up-regulation in both the reference and variant isoforms across all four samples, which emphasizes the possible development of these isoforms as biomarkers for horn cancer. Additionally, 9 out of 14 variants that we selected contain premature termination codon as a result of novel splicing; however, it requires further detailed investigation of effects produced by nonsense-mediated decay of these aberrant transcript on overall expression of the gene, if any.

Since horn cancer has been analysed previously for detection of novel splice variants (Patel et al. 2013), we sought to identify any differences or similarities between results, if any. Surprisingly, none of the previously identified and validated eight splice isoforms were detected in the present study. However, several reasons could be attributed for such an observation. As the previous study was a preliminary study involving small size of dataset without any quality filtering of the data, skipping of all the previous events identified is quite possible. Second, the present study identified and selected a set of novel splice isoforms whose probability of association with horn cancer is much more, as the events were identified in a logical and systematic way after enrichment of genes associated with horn cancer using GO analysis. Third, the software used for assembly (GS de novo assembler vs Cufflinks) and detection of splice events (Blat vs Cuffcompare) was different and it is important to note that full-length transcript assembly is very difficult, which is additionally complicated by artifacts in sample preparation, sequencing and read alignment. The above listed reasons and small-scale analysis without any type of selection criteria of previously generated dataset resulted in skipping of already identified novel splice isoforms. Nonetheless, the findings reported in the present study are absolutely novel and add to existing knowledge about splice isoforms in HC.

The 14 novel splicing events selected for verification might be considered as biomarkers as their potential effect on cancer progression. Consistent down-regulation of both the reference and variant was found for *FOS* gene by RT-qPCR across four HC samples. Alternate 3′ splicing of exon2 in *FOS* resulted in premature stop codon just after position 104 aa culminating in loss of basic leucine zipper DNA-binding domain responsible for specific DNA binding. Although transcripts containing premature stop codon are subjected to nonsense-mediated decay (NMD), it has been studied that stress response elicited in tumour microenvironment leads to inhibition of NMD (Wang et al. 2011). Further studies are required to support such speculation and to study the effect of aberrant expression of this splice isoform on tumour progression. *MAPK9* splice isoform which is a member of MAP kinase family of genes is also called as c-Jun N-terminal kinases (JNK2) involved in proliferation, differentiation, transcription regulation and development (Raciti et al. 2012). *MAPK9* splice isoform exhibited premature stop codon after 293 aa and loss of domain responsible for serine/threonine kinase activity. We observed consistent up-regulation of both the reference and variant for *MAPK9* gene by RT-qPCR across four HC samples. *FGFR1* and *FGFR2* are the members of the fibroblast growth factor receptor (FGFR) family of genes with a variety of roles in cancer (Holzmann et al. 2012). Splice variation in *FGFR1* led to loss of IG1 domain which leads to activation of distinct signalling cascades (Zhang et al. 2006), while splice change observed in *FGFR2* with loss of PTKc domain could not be validated with RT-PCR. Both the genes *FGFR1* and *FGFR2,* reference and variant, were found to be down-regulated by RT-qPCR. *MFAP4* encodes for bovine microfibril-associated protein with probable role in calcium-dependent cell adhesion or intercellular interactions. We also observed intron retention in *HSP90AB1* transcript resulting in premature termination after 380 aa position. *HSP90AB1* plays an important role in folding newly synthesized proteins or stabilizing and refolding denatured proteins after stress (Cheng et al. 2012). *RPS6KB1* splice isoform is part of a protein kinase family involved in the regulation of cellular growth, insulin, and cellular energy. Overexpression of *RPS6KB1* plays an important role in hepatocellular carcinoma (Li et al. 2012) and correlates with adverse prognosis. Similarly, CREBBP (Mullighan et al. 2011), *CRK* (Sriram and Birge 2010), *PTPN6* and *PTPRU* (Hoekstra et al. 2012) genes which might play a probable role in cancer revealed premature termination with loss of domains related to function as shown in Table 2. *SUFU* gene encodes negative regulator of Hedgehog signalling pathway exhibiting signalling cascade that plays a role in pattern formation and cellular proliferation during development. The gene is not well studied in the bovine, but two isoforms with different c terminal have been reported in humans (Tostar et al. 2012). *SUFU* splice isoform identified in this study encodes for longer isoform with different C terminal than reference. *FN1* encodes fibronectin, a glycoprotein present at the cell surface and in extracellular matrix and is involved in cell adhesion and migration processes including embryogenesis, wound healing, blood coagulation, host defense, and metastasis (Goossens et al. 2009). Splice variation of novel exon 25 and skipping of exon 32 revealed in this isoform and resulted in varying length of protein without frameshift. In total, we confirmed expression of these 13 novel splice isoforms, however it remains to be investigated whether these putative protein isoforms are indeed translated in to proteins and in turn functional consequences of these variations. Furthermore, we found an interesting aspect of our analysis of AS events in HC as majority of novel splice isoforms supported by ESTs reported in bovine foetus. This may be due to the activation of genes or isoforms taking part in the foetal development by carcinogenic genes. This observation is supported by an earlier study showing antigenic similarity between bovine horn cancer and foetal tissue (Kuchroo et al. 1979).

## Conclusion

The results reported here are novel in terms of alternative splicing prevailing in HC and have potential implications for intervention of HC at molecular level. Herein, we reported and confirmed several novel splice isoforms differentially expressed in HC which might be useful as biomarkers to diagnose the HC at early stage. Rigorous and cautious filtering of several novel splice isoforms resulted in splice isoforms, most probably associated with HC. In summary, we have identified and validated fourteen putative novel isoforms which are significantly associated with HC and discussed their possible involvement in cancer progression. Further extensive follow-up analysis of novel splice isoforms found in this study might be useful in the development of prospective intervention strategies for HC. The results reported here might also be helpful to understand other cancers prevalent in other species and refine the available information on transcriptome repertoire of bovine species.

## Electronic supplementary material

Below is the link to the electronic supplementary material.

Supplementary material 1 (DOC 196 kb)

Supplementary material 2 (DOC 167 kb)

Supplementary material 3 (DOC 49 kb)

## Abbreviations

HC: Horn cancer
HN: Horn normal
AS: Alternative splicing
EST: Expressed sequence tag
KEGG: Kyoto encyclopedia of genes and genomes
DAVID: Database for annotation, visualization and integrated discovery
BLAT: Blast-like alignment tool
IGV: Integrated genome viewer
GO: Gene ontology
NMD: Nonsense-mediated decay
