# Supplementary material for: Identification and quantification of novel RNA isoforms in horn cancer of Bos indicus by comprehensive RNA-Seq
Source: 3 Biotech. 2016 Dec 7;6(2):259. doi: 10.1007/s13205-016-0577-5 (PMC5143338; doi:10.1007/s13205-016-0577-5)
Supplement: Supplementary file 1 — Supplementary material 1 (DOC 196 kb) [file 13205_2016_577_MOESM1_ESM.doc]

Table ST1: List of isoforms with significant differential expression in horn cancer

| **Gene** | **Chr.** | **FPKM Normal** | **FPKM Cancer** | **Fold Change** | **p_value** |
| --- | --- | --- | --- | --- | --- |
| KRT6B | chr5:30263838-30269331 | 0.906101 | 2259.19 | 11.2838 | 5.87E-13 |
| KRT6A | chr5:30244904-30250158 | 0.91142 | 2135.02 | 11.1938 | 6.57E-13 |
| CA1 | chr14:75921271-75980972 | 1.08583 | 335.882 | 8.27301 | 2.01E-05 |
| KRT14 | chr19:42831349-42835714 | 25.6826 | 6037.56 | 7.87703 | 2.35E-14 |
| CA2 | chr14:75773520-75789877 | 2.8833 | 249.65 | 6.43604 | 8.78E-09 |
| TNS4 | chr19:41642809-41666190 | 0.772651 | 62.2431 | 6.33195 | 1.13E-08 |
| SAA3 | chr29:27517799-27521530 | 4.76094 | 377.878 | 6.31053 | 9.92E-06 |
| CDH3 | chr18:34862140-34908973 | 1.12133 | 62.8269 | 5.8081 | 5.02E-06 |
| KRT17 | chr19:42873493-42885142 | 78.6113 | 2630.95 | 5.0647 | 7.87E-05 |
| RCAN1 | chr1:219330-339078 | 6.88752 | 215.732 | 4.96912 | 1.37E-04 |
| KRT5 | chr5:30227338-30233594 | 76.2955 | 2365.66 | 4.9545 | 2.97E-04 |
| KIF20A | chr7:48903333-48911736 | 0.547259 | 16.6264 | 4.9251 | 5.75E-04 |
| ANGPTL2 | chr11:100987666-101285826 | 1.95783 | 53.4278 | 4.77026 | 2.46E-05 |
| LAMB3 | chr16:71690550-71733413 | 2.24974 | 55.5047 | 4.62478 | 4.35E-04 |
| INHBA | chr4:81627089-81643152 | 1.42303 | 34.4126 | 4.5959 | 1.53E-06 |
| PERP | chr9:78632483-78646816 | 15.4477 | 372.081 | 4.59015 | 1.12E-06 |
| S100A9 | chr3:18278375-18281115 | 8.19194 | 189.4 | 4.53109 | 4.66E-04 |
| TFAP2C | chr13:59957873-59967430 | 1.35289 | 29.6694 | 4.45486 | 3.25E-05 |
| TRIM29 | chr15:29104436-29132017 | 7.26011 | 133.04 | 4.19572 | 1.67E-04 |
| FABP5 | chr14:41654252-41661034 | 9.10851 | 155.523 | 4.09377 | 4.26E-04 |
| GAPDH | chr5:110079397-110083681 | 2.54202 | 42.6897 | 4.06984 | 1.14E-03 |
| ITGA6 | chr2:24946045-25033336 | 7.94967 | 129.515 | 4.02608 | 1.21E-04 |
| SLC7A8 | chr10:21864839-21917890 | 1.46039 | 22.6584 | 3.95563 | 2.65E-04 |
| SLC16A1 | chr3:32652385-32681849 | 4.64884 | 70.5645 | 3.924 | 1.65E-05 |
| NDRG1 | chr14:7517146-7573315 | 28.1473 | 415.462 | 3.88365 | 1.18E-04 |
| GJA1 | chr9:31507203-31520216 | 32.9129 | 459.039 | 3.80189 | 3.82E-05 |
| FAM84A | chr11:86149107-86154086 | 1.61064 | 22.3395 | 3.79389 | 5.43E-04 |
| GNA15 | chr7:19302086-19321124 | 1.87126 | 25.6416 | 3.77641 | 5.58E-04 |
| CASP3 | chr27:16297097-16322281 | 2.13109 | 28.6941 | 3.75109 | 9.87E-04 |
| DSC3 | chr24:26837284-26892201 | 3.4292 | 44.6015 | 3.70115 | 1.60E-04 |
| GNG12 | chr3:82571701-82573478 | 2.38387 | 30.4872 | 3.67683 | 7.10E-04 |
| C1QTNF6 | chr5:80750719-80757995 | 1.64053 | 20.3772 | 3.63472 | 8.65E-04 |
| ANXA8L1 | chr28:41711799-41728108 | 26.9611 | 333.412 | 3.62835 | 1.26E-03 |
| PPARD | chr23:9590564-9679593 | 2.20879 | 26.5943 | 3.58979 | 3.66E-04 |
| SHMT2 | chr5:60431869-60439435 | 5.11228 | 60.6774 | 3.56912 | 4.94E-04 |
| EIF4EBP1 | chr27:35254185-35277532 | 5.90103 | 69.6994 | 3.56211 | 1.17E-03 |
| PLAUR | chr18:51520455-51533746 | 6.52434 | 75.9572 | 3.54128 | 4.64E-04 |
| FAM57A | chr19:21946325-21955278 | 1.83711 | 21.381 | 3.54082 | 1.16E-03 |
| SLC7A1 | chr12:30668296-30699840 | 4.28748 | 48.1545 | 3.48947 | 1.40E-03 |
| TP63 | chr1:78833387-78834038 | 20.891 | 232.627 | 3.47707 | 1.92E-04 |
| SLC2A1 | chr3:109256164-109290327 | 5.18999 | 54.2644 | 3.3862 | 2.56E-04 |
| S100A14 | chr3:17931476-17933537 | 22.0022 | 221.539 | 3.33184 | 5.37E-05 |
| H1F0 | chr5:116099257-116101520 | 9.19041 | 91.551 | 3.31637 | 3.83E-04 |
| BNIP3 | chr26:50799308-50805625 | 10.4377 | 101.716 | 3.28468 | 1.35E-03 |
| ARL4C | chr3:120442822-120446835 | 3.46317 | 31.5046 | 3.18539 | 3.46E-04 |
| RRS1 | chr14:30474503-30476267 | 3.76859 | 34.2507 | 3.18404 | 1.07E-03 |
| TRPS1 | chr14:46278926-46282180 | 3.69111 | 33.4582 | 3.18023 | 4.60E-04 |
| PPP1R14C | chr15:11854755-11856893 | 4.43592 | 39.7268 | 3.16281 | 6.90E-04 |
| ASS1 | chr11:104306221-104358267 | 16.794 | 139.39 | 3.05311 | 2.29E-04 |
| TFRC | chr1:71652444-71682290 | 6.81137 | 54.5144 | 3.00062 | 4.91E-04 |
| PVRL1 | chr15:28625423-28697683 | 7.3835 | 57.1131 | 2.95145 | 2.27E-04 |
| LSAMP | chr1:61346177-62195699 | 15.8815 | 121.743 | 2.93842 | 1.56E-03 |
| MMP13 | chr15:4611275-4623038 | 19.7454 | 133.573 | 2.75804 | 1.04E-03 |
| ADK | chr15:33916980-33918811 | 8.25713 | 55.0851 | 2.73795 | 1.47E-03 |
| PRELP | chr16:235763-242194 | 64.0366 | 11.0258 | -2.53802 | 1.54E-03 |
| CTSS | chr3:21313969-21336898 | 79.4562 | 13.277 | -2.58123 | 1.26E-03 |
| RHOB | chr11:80693207-80695420 | 246.145 | 39.5981 | -2.636 | 1.29E-03 |
| ZFP36 | chr18:48495189-48496561 | 321.436 | 47.8504 | -2.74793 | 7.77E-04 |
| BLA-DQB | chr23:26092956-26100872 | 175.774 | 23.0227 | -2.93259 | 5.99E-04 |
| MIR628 | chr10:55522364-55561669 | 19.0403 | 2.46435 | -2.94978 | 1.64E-03 |
| SOX2 | chr1:86957653-86959874 | 37.9688 | 4.77609 | -2.99091 | 1.07E-03 |
| CYR61 | chr3:61679495-61682405 | 547.385 | 68.3933 | -3.00063 | 8.52E-04 |
| C3 | chr7:16320827-16356820 | 74.1274 | 9.16153 | -3.01635 | 3.34E-04 |
| TGFBR2 | chr22:5018656-5110524 | 64.3636 | 7.38882 | -3.12283 | 1.35E-03 |
| TSC22D1 | chr12:13650135-13778208 | 215.332 | 23.6634 | -3.18583 | 1.25E-03 |
| MGST1 | chr5:99796499-99819492 | 210.124 | 22.4689 | -3.22524 | 1.64E-04 |
| OAS1Y | chr17:64353850-64387639 | 65.4385 | 6.82504 | -3.26123 | 6.11E-04 |
| ACSS3 | chr5:12758312-12989404 | 24.4374 | 2.51035 | -3.28313 | 4.32E-04 |
| LITAF | chr25:11372738-11408870 | 52.4801 | 5.22887 | -3.3272 | 3.62E-04 |
| CALCOCO2 | chr19:38575521-38599150 | 69.5315 | 6.89306 | -3.33445 | 8.03E-05 |
| ALDH2 | chr17:65369433-65394967 | 57.04 | 5.58611 | -3.35206 | 1.39E-03 |
| GNA12 | chr25:42439689-42511685 | 31.397 | 3.0531 | -3.36228 | 1.70E-03 |
| METTL7A | chr5:31932670-31943736 | 29.7932 | 2.86437 | -3.3787 | 5.29E-04 |
| JAM2 | chr1:10014214-10096812 | 34.1064 | 3.26478 | -3.38498 | 5.97E-04 |
| CFB | chr23:26198254-26204281 | 63.9949 | 5.83826 | -3.45435 | 1.14E-04 |
| PDPN | chr16:51337973-51374400 | 69.1854 | 6.1392 | -3.49435 | 7.34E-05 |
| JUN | chr3:92701551-92704977 | 376.762 | 33.055 | -3.51071 | 2.51E-04 |
| ZFP36L2 | chr11:26935517-26939771 | 55.808 | 4.87352 | -3.51744 | 4.04E-05 |
| KRT7 | chr5:30520141-30534152 | 61.2406 | 5.23656 | -3.5478 | 4.50E-04 |
| IFI6 | chr2:131115858-131119447 | 300.026 | 25.2214 | -3.57237 | 1.80E-04 |
| AOC3 | chr19:43890544-43897416 | 32.4249 | 2.70688 | -3.5824 | 1.74E-04 |
| HERC6 | chr6:37041982-37095466 | 18.4783 | 1.51593 | -3.60756 | 4.38E-04 |
| IER2 | chr7:10700758-10702534 | 227.732 | 18.4327 | -3.627 | 3.62E-05 |
| CAT | chr15:64650470-64686447 | 51.6942 | 4.15785 | -3.63609 | 9.97E-05 |
| ATP1B1 | chr16:34167862-34441364 | 60.4237 | 4.6047 | -3.71393 | 1.55E-04 |
| EFEMP1 | chr11:39994378-40063931 | 51.514 | 3.86657 | -3.73584 | 2.28E-04 |
| LY6E | chr14:1392676-1396381 | 173.742 | 12.2644 | -3.8244 | 6.72E-05 |
| CDH13 | chr18:8251029-9276280 | 27.8706 | 1.93655 | -3.84718 | 1.11E-04 |
| PPAP2B | chr3:95179449-95223720 | 114.108 | 7.80604 | -3.86967 | 2.82E-05 |
| TXNIP | chr3:23063039-23067196 | 175.795 | 11.8453 | -3.89151 | 6.52E-06 |
| METTL7A | chr5:31833925-31844990 | 27.8653 | 1.87063 | -3.89688 | 1.71E-04 |
| RAPGEF3 | chr5:35634360-35655920 | 18.8445 | 1.2442 | -3.92085 | 1.59E-03 |
| GREM1 | chr10:29418496-29433023 | 17.8093 | 1.12278 | -3.98748 | 7.92E-05 |
| SEPP1 | chr20:33675454-33685129 | 116.253 | 6.93541 | -4.06714 | 2.88E-06 |
| EPAS1 | chr11:29881365-29975241 | 309.337 | 18.2999 | -4.07927 | 2.29E-04 |
| TUBA1D | chr2:113142452-113148428 | 123.972 | 7.19467 | -4.10695 | 2.69E-04 |
| C7 | chr20:35557085-35614137 | 23.7588 | 1.32828 | -4.16084 | 2.59E-04 |
| AQP3 | chr8:79407538-79413493 | 58.6414 | 3.26641 | -4.16614 | 7.57E-05 |
| TSPAN12 | chr4:87807815-87873187 | 16.6136 | 0.884676 | -4.23107 | 7.03E-04 |
| AQP1 | chr4:67552059-67566279 | 215.881 | 11.4279 | -4.2396 | 1.80E-04 |
| DUSP1 | chr20:4566745-4581710 | 248.847 | 12.9375 | -4.26563 | 2.26E-05 |
| EPHX1 | chr16:25868912-25906903 | 73.022 | 3.52647 | -4.37204 | 1.55E-05 |
| EPCAM | chr11:30983870-30994307 | 71.708 | 3.46105 | -4.37285 | 8.47E-06 |
| LYZ | chr5:47928100-47936936 | 23.4975 | 1.11251 | -4.40063 | 3.94E-04 |
| F3 | chr3:51781195-51792080 | 142.716 | 6.30192 | -4.50121 | 1.14E-06 |
| CH25H | chr26:11431465-11433524 | 51.0153 | 2.20084 | -4.53481 | 1.65E-05 |
| EGR1 | chr7:49116638-49122582 | 309.447 | 11.1845 | -4.79012 | 1.03E-05 |
| APOD | chr1:73019924-73034153 | 373.494 | 13.4323 | -4.7973 | 3.27E-06 |
| VCAM1 | chr3:45238683-45260995 | 40.9822 | 1.41202 | -4.85916 | 2.63E-06 |
| CETN2 | chrX:22715988-22720105 | 60.9209 | 2.09844 | -4.85955 | 8.11E-05 |
| KRT8 | chr10:75127860-75129640 | 165.208 | 5.64346 | -4.87156 | 8.25E-07 |
| PPP1R15A | chr18:55252427-55256323 | 124.352 | 4.07757 | -4.93058 | 7.87E-06 |
| CYYR1 | chr1:9186391-9302492 | 22.5112 | 0.736901 | -4.93303 | 7.76E-04 |
| NFIB | chr8:31596106-31849365 | 24.8224 | 0.782764 | -4.98692 | 5.07E-05 |
| CXCL12 | chr28:45096040-45111279 | 19.4777 | 0.598683 | -5.02389 | 5.21E-04 |
| IGJ | chr6:88854438-88863836 | 109.939 | 3.34977 | -5.0365 | 7.03E-07 |
| FZD4 | chr29:8868173-8877446 | 26.7221 | 0.81034 | -5.04336 | 8.13E-07 |
| TSPAN13 | chr4:26015622-26053688 | 71.3493 | 2.16137 | -5.04488 | 1.64E-06 |
| LMO2 | chr15:64142873-64155512 | 62.4702 | 1.88062 | -5.05389 | 1.71E-04 |
| FAM107A | chr22:43516811-43539870 | 57.5301 | 1.60888 | -5.16019 | 2.08E-04 |
| ADRA2A | chr26:31950278-31954532 | 17.8032 | 0.454448 | -5.29187 | 1.48E-03 |
| CITED2 | chr9:79820257-79822668 | 78.13 | 1.87782 | -5.37875 | 3.77E-07 |
| RNASE4 | chr10:25707885-25729656 | 82.7376 | 1.96797 | -5.39376 | 7.56E-05 |
| GP2 | chr25:19159168-19175597 | 36.6143 | 0.762113 | -5.58626 | 7.70E-04 |
| BPIFA1 | chr13:63557091-63564155 | 4950.95 | 74.6958 | -6.05053 | 2.85E-10 |
| TPPP3 | chr18:33891866-33896764 | 136.576 | 2.04387 | -6.06225 | 8.71E-04 |
| FOS | chr10:88614119-88617575 | 894.044 | 7.43881 | -6.90913 | 8.41E-06 |
| FOSB | chr18:52773546-52782085 | 100.514 | 0.734443 | -7.09654 | 2.07E-04 |
| CXCL17 | chr18:51140350-51146972 | 247.399 | 1.64794 | -7.23003 | 1.52E-05 |
| BPIFB1 | chr13:63591797-63615377 | 269.145 | 0.975227 | -8.10843 | 1.21E-05 |
