# Supplementary material for: Identification and quantification of novel RNA isoforms in horn cancer of Bos indicus by comprehensive RNA-Seq
Source: 3 Biotech. 2016 Dec 7;6(2):259. doi: 10.1007/s13205-016-0577-5 (PMC5143338; doi:10.1007/s13205-016-0577-5)
Supplement: Supplementary file 2 — Supplementary material 2 (DOC 167 kb) [file 13205_2016_577_MOESM2_ESM.doc]

Table ST2: Details of selected novel splice variants evaluated for type of splice event and corresponding protein change

| **Gene** | **EST support** | **Variation** | **Chr** | **Change at protein sequence** |
| --- | --- | --- | --- | --- |
| **Selected novel splice variants only present in horn cancer** | | | | |
| *MSH6* | yes | alternate 5' splicing exon 2 | 11 | Addition of S after 85 position without frameshift |
| *MAGED1* | Yes | novel 5' exon, skipping of exon 1 | X | No protein change |
| *HSP90AB1* | Yes | intron tetention between exon 7-8 | 23 | premature stop codon after 380 aa, HSP90 domain lost, primer designed |
| *RIPK1* | Yes | novel 5' exon | 23 | No Protein change |
| *CTNNB1* | Yes | alternate 5' splicing exon 16 | 22 | No protein change |
| *RAF1* | Yes | skipping of exon 8 | 22 | primer designed |
| *JUP* | Yes | novel 3' exon | 19 | No Protein change |
| *PRKAR1A* | Yes | novel 5' exon | 19 | No Protein change |
| *STAT3* | Yes | alternate 3' splicing exon 21 | 19 | deletion of aa S at position 701 |
| *PLCG1* | Yes | alternate 5' splicing exon 32 | 13 | deletion of aa at position 1216 |
| *FOS* | Yes | alternate 3' splicing exon 2 | 10 | premature stop codon after 104 aa, primer designed |
| *PSEN1* | Yes | novel 5' exons(2) | 10 | No protein change, 5' UTR change |
| *MAPK9* | Yes | alternate 5' splicing exon 10 | 7 | premature stop codon after 293 aa, primer designed |
| *CAMK2D* | Yes | skipping of exon 14 | 6 | deletion of 328-338 aa without frameshift |
| *NFKB1* | Yes | alternate 5' exon,skipping of exon 1-2 | 6 | Alternate 3' UTR without change in protein |
| *PPP3CA* | Yes | skipping of exon 13 | 6 | deletion of 448-457 aa without frameshift |
| *IRAK4* | Yes | alternate 5' exon | 5 | Alternate 5' UTR without change in protein |
| *SHC1* | Yes | alternate 5' splicing exon 11,intron retention between exon 10-11 | 3 | additional aminoacid between 307-308,similar to yak |
| *RALB* | Yes | alternate 5' exon, skipping of exon 1-2 | 2 | Alternate 5' UTR without change in protein |
| *GSK3B* | Yes | skipping of exon 9 | 1 | deletion of 304-316 aa without frameshift |
| *IL1RAP* | Yes | novel 3' exons(3) | 1 | Alternate 3' UTR without change in protein |
| *NCK1* | Yes | alternate 5' exon,alternate 5' splicing exon 2 | 1 | Alternate 5' UTR without change in protein |
| *SGK1* | Yes | novel 5' exon | 9 | Alternate 5' UTR without change in protein |
| *SGK1* | Yes | novel 5' exon | 9 | Alternate 5' UTR without change in protein |
| *CAPN1* | No | alternate 5' exon | 29 | Alternate 5' UTR without change in protein |
| *FGFR1* | No | novel 5' exon,skipping of exon 2, alternate 3' splicing exon 3 | 27 | deletion of aa 31-117, loss of Ig1_FGFR domain, Primer designed |
| *FGFR2* | No | skipping of exon 3,alternate exon 8,skipping of exon 10-13,alternate 3' splicing exon 9 | 26 | deletion of aa 448-639, loss of PTKc_FGFR domain, Primer designed |
| *MAPK3* | No | alternate 5' exon | 25 | Alternate 5' UTR without change in protein |
| *RIPK1* | No | novel 5' exon | 23 | Alternate 5' UTR without change in protein |
| *IGF1R* | No | novel exon between 5-6 | 21 | present at the end of read |
| *MFAP4* | No | novel 5' exon,skipping of exon 2-5,alternate 5' splicing exon 6 | 19 | premature stop codon after 157 aa, primer designed, domain lost |
| *RPS6KB1* | No | alternative 3' splicing exon 7 | 19 | premature stop codon after 234 aa, Part of STKc domain lost, primer designed |
| *CCNE1* | No | novel 5' exon | 18 | Alternate 5' UTR without change in protein |
| *ILK* | No | intron retention between exon 6-7 | 15 | insertion of 33 aa after 177 aa without frameshift |
| *PTK2* | No | skipping of exon 3,alternate 5' splicing exon 22 | 14 | premature stop codon after 624 aa, domain lost |
| *SRC* | No | novel 5' exons(2) | 13 | Alternate 5' UTR without change in protein |
| *PAK6* | No | alternate splicing of exon 10 | 10 | Alternate 3' UTR without change in protein |
| *MAP3K7* | No | alternate 3' splicing exon 1,novel exon between 12-13 | 9 | insertion of 27 aa after 403 aa without frameshift |
| *IFNAR1* | No | intron retention between exon 4-5 | 1 | present at the end of read |
| *PIK3CB* | No | skipping of exon 2 | 1 | Alternate 5' UTR without change in protein |
| *SGK1* | No | novel 5' exons(2) | 9 | additional in-frame exons at the 5' end compared to Ref resulting in isoform with a longer and an unique N-terminus compared to ref |
| *MAP2K4* | No | skipping of exon 2 | 19 | deletion of 41-52 aa without frameshift |
| **Selected novel splice variants only present in horn normal** | | | | |
| *AKT2* | yes | alternate 5' exon | 9 | Alternate 3' UTR without change in protein |
| *ARHGEF6* | No | alternate 5' exon | 1 | Alternate 5' UTR without change in protein |
| *CAMK2D* | yes | alternate 5' splicing exon 13, skipping of exon 14 | 26 | Alternate 5' UTR without change in protein |
| *CAPN1* | yes | alternate 3' splicing exon 1 | 17 | Alternate 3' UTR without change in protein |
| *CDC42* | No | alternate 5' exon | 22 | Alternate 3' UTR without change in protein |
| *CDC42EP1* | No | alternate 5' exon | 6 | Alternate 5' UTR without change in protein |
| *CREBBP* | No | alternate 3' splicing exon 9 | 1 | Alternate 5' UTR without change in protein |
| *CRK* | yes | novel exon between 1-2, alternate 3' splicing of exon 2 | 20 | Alternate 5' UTR without change in protein |
| *CTNNB1* | yes | alternate 3' exon | 6 | deletion of 448-457 aa without frameshift |
| *ERBB3* | No | intron retention between exon 8-9 | 10 | Alternate 5' UTR without change in protein |
| *FGF7* | No | novel 5' exon | 5 | premature stop codon after 457 aa, primer designed, Part of PTPc domain lost |
| *FGFR1* | yes | novel 5' exon | 23 | Alternate 5' UTR without change in protein |
| *FGFR1* | yes | skipping of exon 2,novel 5' exon | 3 | Addition of 9 aa after 771 without frameshift |
| *FGFR1* | No | alternate 3' splicing exon 3 | 9 | Addition of A and 6 aa after 732 and 946 aa without frameshift |
| *FGFR2* | yes | alternate 3' splicing exon 9, alternate exon 8 | 2 | premature stop codon after 312 aa, primer designed, several domains of fibronectine and PTPc are lost |
| *FGFR2* | yes | skipping of exon 3 | 2 | Alternate 5' UTR without change in protein |
| *FOS* | yes | 5' alternate exon and 3' alternate splicing of exon 2 | 11 | deletion of 69-106 aa without frameshift |
| *FOSB* | No | 3' alternate exon | 25 | premature stop codon after 670 aa, no domain loss |
| *FYN* | yes | novel 3' exons | 10 | Addition of 11 aa after 175 without frameshift |
| *IFNAR2* | No | alternate 5' exon | 19 | Alternate 5' UTR without change in protein |
| *ITPRIP* | yes | alternate 5' exon | 26 | Same size of prtein with different terminal 52 aa, different C terminal domain than Ref |
| *MAPK1* | yes | novel 3' exon | 9 | Alternate 5' UTR without change in protein |
| *MAPKAPK3* | yes | alternate 3' splicing exon 1 | 21 | deletion of 1022-1052 aa without frameshift |
| *NFKB1* | yes | alternate 5' exon | 16 | Truncated protein of 1719 aa without loss of conserved domain |
| *PIK3CA* | No | novel 5' exon, alternate 5' splicing exon 1 | 13 | deletion of aa at position 101 without frameshift |
| *PIK3R1* | No | Novel 5' exon | 9 | Alternate 3' UTR without change in protein |
| *PPP3CA* | yes | skipping of exon 13 | 1 | Alternate 5' UTR without change in protein |
| *PSEN1* | yes | novel 5' exons(2),skipping of exon 8 | 26 | Alternate 5' UTR without change in protein |
| *PTPN6* | No | alternate 5' exons (3), skipping of exon 12 | 17 | Alternate 3' UTR without change in protein |
| *PTPRA* | yes | novel exon between 1-2 | 22 | Alternate 3' UTR without change in protein |
| *PTPRF* | No | novel exon between 13-14 | 6 | Alternate 5' UTR without change in protein |
| *PTPRK* | No | alternate 5' splicing of exon 14, skipping of exon 19 | 1 | Alternate 5' UTR without change in protein |
| *PTPRU* | No | alternate 5' splicing exon 7, skipping of exon 15 | 20 | Alternate 5' UTR without change in protein |
| *RALB* | yes | alternate 5' exon | 6 | deletion of 448-457 aa without frameshift |
| *RAPGEF1* | yes | skipping of exon 3 | 10 | Alternate 5' UTR without change in protein |
| *SH2B1* | yes | novel exon between 7-8 | 5 | premature stop codon after 457 aa, primer designed, Part of PTPc domain lost |
| *SMOC1* | No | alternate 5' splicing exon 6 | 23 | Alternate 5' UTR without change in protein |
| *STAT5B* | yes | alternate 5' exon | 3 | Addition of 9 aa after 771 without frameshift |
| *SUFU* | No | alternate 5' splicing exon 7,novel 3' exons | 9 | Addition of A and 6 aa after 732 and 946 aa without frameshift |
| *TAB2* | No | alternate 5' exon, alternate 5' splicing exon 1 | 2 | premature stop codon after 312 aa, primer designed, several domains of fibronectine and PTPc are lost |
| *TP53BP1* | yes | skipping of exon 17 | 2 | Alternate 5' UTR without change in protein |
| *TPR* | No | alternate splicing exon 36 | 11 | deletion of 69-106 aa without frameshift |
| *YWHAB* | yes | alternate 5' splicing exon 3 | 25 | premature stop codon after 670 aa, no domain loss |
| **Selected novel splice variants only present in both horn cancer and normal** | | | | |
| *THBS2* | Yes | Intron retention | 9 | Alternate 3' UTR without change in protein |
| *DUSP7* | Yes | 3' terminal exon skipping, intron retention | 22 | Alternate 5' UTR without change in protein |
| *LIMK2* | yes | Alternate 3' UTR | 17 | Alternate 3' UTR without change in protein |
| *JUP* | Yes | Novel 5' and 3' exon | 19 | Alternate 5' and 3' UTR without change in protein |
| *FN1* | No | skipping of exon 32 | 2 | deletion of 1631-1720 aa without frameshift primers designed |
| *CTNNB1* | yes | Alternate 3' UTR | 22 | Alternate 3' UTR without change in protein |
| *FN1* | yes | novel exon 25 | 2 | Addition of 91 aa after 1266 without frameshift primers designed |
| *VCL* | yes | skipping of exon 19 | 28 | deletion of 916-983 aa without frameshift |
| *MAPK1* | yes | Novel 3' exon | 17 | Alternate 3' UTR without change in protein |
| *FOXO3* | yes | novel 3' exon | 9 | Alternate 3' UTR without change in protein |
| *RAC1* | yes | alternate 3' splicing exon 1 | 25 | Alternate 3' UTR without change in protein |
| *MYL12A* | yes | skipping of exon 2 | 24 | Alternate 5' UTR without change in protein |
| *PPP3R1* | No | novel 5' exon | 11 | Alternate 5' UTR without change in protein |
| *MAX* | yes | skipping of exon 2, alternate 5' splicing exon 4 | 10 | deletion of 13-21 and 59-60 aa without frameshift |
| *BCL2L1* | yes | alternate 5' exon | 13 | Alternate 5' UTR without change in protein |
| *STAT1* | yes | alternate 3' (2) and 5' exons | 2 | Alternate 3' and 5' UTR without change in protein |
| *PPM1B* | yes | alternate 3' exon | 11 | Alternate 3' UTR without change in protein |
