# Supplementary material for: Identification and quantification of novel RNA isoforms in horn cancer of Bos indicus by comprehensive RNA-Seq
Source: 3 Biotech. 2016 Dec 7;6(2):259. doi: 10.1007/s13205-016-0577-5 (PMC5143338; doi:10.1007/s13205-016-0577-5)
Supplement: Supplementary file 3 — Supplementary material 3 (DOC 49 kb) [file 13205_2016_577_MOESM3_ESM.doc]

Table ST3: List of primer sequences designed for amplification of splice variant and corresponding reference transcript in horn cancer

| **Sr. No.** | **Name of Gene** | **Primer Sequence (5’-3’)** | **Product Size** |
| --- | --- | --- | --- |
| 1 | *FOS ref* | CGTCAATGCGCAGGACTACT  GGAGACTAGGGTGGGCTGTA | 121 |
|  | *Fos variant* | CGTCAATGCGCAGGACTACT  TTCTGGGGACAACATTGTAGGT | 108 |
| 2 | *MAPK9 ref* | CAACCTTCAGATGCAGCAGT  ATCATCGACAGCCTTCCAGG | 136 |
|  | *MAPK9 variant* | ACCTTCAGTATAGGAAACTGCAA  TGCTGCTTACTGCTGCATCT | 154 |
| 3 | *FGFR1 ref* | GCTCCGCGAGTCAGCTTG  AAGAGGAGACACTTCCGGCT | 122 |
|  | *FGFR1 variant* | CCGGAGCAAGTCTCAGATGC  GCACTGCGTGCAGTTTCTTT | 163 |
| 4 | *FGFR2 ref* | ACTCGCCTCTCTTCAACTGC  TTTGCCCAGCGTCAGCTTAT | 111 |
|  | *FGFR2 variant* | AGACAGTGCATTCATCGAGAT  AGTCGGCCATTTGTGGTCTT | 137 |
| 5 | *MAFP4 ref* | AACATGCACCTGCTGACACT  GCTGACAGCATTGGGTGAGA | 120 |
|  | *MAFP4 variant* | AACATGCACCTGCTGACACT  TCAGCGAGTCGCTTGGCAA | 95 |
| 6 | *HSP90AB1 ref* | CGGAATATCTCAACTTCATCCGT  TCAGAGAAGAGCTCAAGGCAC | 142 |
|  | *HSP90AB1 var* | TCCGGAATATCTCAGTGAGTATCTT  TACAGGAGTCGCCGGGATAA | 193 |
| 7 | *RPS6KB1 ref* | ATCACCAAGGTCACGTCAAAC  CAAGATCTCAGGGGCCATGTA | 116 |
|  | *RPS6KB1 var* | ATCACCAAGGTGGAGATATACTGT  CAAGATCTCAGGGGCCATGTA | 144 |
| 8 | *CREBBP ref* | GTCTGCCAACAGCAGGGATG  GGCTGGTTACCCAAGATGCC | 123 |
|  | *CREBBP var* | AACAGCAGGGTGGGATGAATA  GCTGGTTACCCAAGATGCCT | 119 |
| 9 | *CRK ref* | GTCAACACTCCGCTCCCTAA  AGCTCACCGACCTCCAAAG | 113 |
|  | *CRK var* | GAATGCGGAGGACAGCGAA  TCACCGACCTCCAATCAGA | 106 |
| 10 | *PTPN6 ref* | GTGAGCCTGGGGGTGTTC  GATGCCGGCGCTGCAATG | 101 |
|  | *PTPN6 var* | GTGAGCCTGGGGGTGTTC  ACAGTCCAGGCCTGCAAT | 102 |
| 11 | *PTPU ref* | GTAAAGGAGCCCCCTACCCC  CCGTCGCCAATGATGGAGTT | 101 |
|  | *PTPU var* | AGCTGATCGTAAAGGCTGCT  CCGTCGCCAATGATGGAGTT | 80 |
| 12 | *SUFU ref* | GTTACAAAGCCAGGCAGCTC  CGTTGTGATGCAACTGTGGG | 95 |
|  | *SUFU var* | TGGTTACAAATTCTGTTGACCGA  TGGGAAGTTTGAATTCCTCTGGA | 91 |
| 13 | *FN1 ref1* | CGAGCCCTGAGGATGGAATC  CAGGAATGGCTGTGGACTGG | 171 |
|  | *FN1 var1* | CAGCCCACAGTGGAGTATGT  TTGGTGCAGGAATGGTGGTT | 100 |
| 14 | *FN1 ref2* | GAAGTGGTCCATGCCGATCA  GAGGGACAGCTGGGATGATG | 39 |
|  | *FN1 var2* | CAGTAGTTGCGGCAGGAGAA  GAGGGACAGCCGTTTGTTGT | 77 |
